# The Disorders of Speech

**Published:** 1895-09

**Authors:** 


*The Disorders of Speech.* By JOHN WYLLIE, M.D. Pp. viii., 495. Edinburgh: OLIVER AND BOYD. [1894.]

This work not only deals with the disorders of speech, but also with the nature and origin of speech itself. It forms a complete study of the faculty of speech, and of its pathological changes. It is original in its conception and in the way in which this has been carried out, and should place the author amongst the most distinguished of medical writers. Its superlative qualities will, we think, cause it to be regarded as a classic in medical literature. This is high praise, but is due to the solid merits of the work and to the lucidity of the author's prose. Dr. Wyllie has evidently devoted many years of hard work and close thinking to the study of speech disorders, and has thus attained a complete mastery of his subject. The book bears evidence of the writer's wide experience, skilled powers of clinical observation, and full acquaintance with the literature of the subject. Thus, by clinical experience and extensive reading the author has thoroughly qualified himself for his work. But from painful experience in medical literature, we are only too well aware that a writer may have extensive knowledge without the gift of clearly presenting his facts to the reader. In this book, however, the style is never obscure and never involved; under Dr. Wyllie's skilful guidance, the difficulties of the subject, in many respects a most intricate and difficult one, disappear, and all seems plain and simple to the reader. It is only when the end of the book, in which there is not a dull page from cover to cover, is reached that we appreciate the magnitude of the task, and the labour that it must have involved to accomplish so successful a result.

The author begins by considering the subject of stammering, and in no section is the stamp of originality which characterises the whole work more manifest than this one. Many of our readers will be already conversant with the physiological alphabet that he has devised for the study and treatment of these cases. The proper treatment of stammering is too much neglected by the medical practitioner; and those who wish for a more accurate knowledge of it, and for a successful method of treatment based on scientific principles, cannot do better than to become thoroughly conversant with the author's writings.

Dr. Wyllie also shows how his physiological alphabet may be modified and adapted for the oral training of deaf mutes. He rightly emphasises the superiority of the oral system of teaching over all others, for these unfortunate persons; and thinks that the reason why it has never come into such general use in England as in France and Germany lies in the difficulties inherent in the English language, especially in the spelling, which is no longer even approximately phonetic. The reader will here find instruction as to the way in which these difficulties may be met.

Hysterical aphonia is fully considered, and in connection with this subject the author brings forward good evidence for his views, opposed to what is generally taught, that the larynx is concerned in whispering. Hysterical mutism is regarded as a more severe degree of hysterical aphonia, the larynx being the part affected in both. For a useful classification of the varieties of stammering, and of hysterical mutism, based on the same lines respectively, we must refer the reader to the book itself. A most interesting account of the other chief functional disorders of speech closes the first part of the book.

The second part deals with the development of speech in the child, which is treated at some length; full reference being made to the important work that has been done on this subject, especially to Preyer's book on *The Mind of the Child*. The consideration of the developmental derangements of speech follows naturally upon this, and the whole section is dealt with in a fascinating way. Defects of speech in idiots and imbeciles, dumbness or defective speech in persons not obviously imbecile, form the subjects of Chapters V. and VI. With regard to the latter persons, Dr. Wyllie thinks that, though not obviously imbecile, there is evidence of weakness of mind in these cases.

An excellent summary of the chief views as to the origin and development of language, the origin of writing and of printing, and then a valuable chapter on the disorders of speech in the various forms of insanity, follow.

By the foregoing considerations the author has paved the way and prepared the reader for what is perhaps the most important part of his subject, and certainly the most difficult part of it to treat well and lucidly—namely, aphasia. So much has been written about aphasia in its different forms, and such an abundant superstructure of theory has been raised upon the actual verified facts, that considerable judgment and discrimination are necessary to deal satisfactorily with it. This soundness of judgment the author possesses in signal measure, and the result is a masterly delineation of the subject most valuable to anyone desirous of acquiring a good working knowledge of the different varieties of aphasia.

The study of aphasia has thrown much light upon the workings of the mind, and more is known of the mental processes concerned in speech than of any other mental operation, but there are still many gaps in our knowledge. We can for instance represent, as the author points out, the manner in which, by combination of the impressions reaching the brain through the several afferent channels of the special senses, the idea of a concrete object such as an apple is built up; but we cannot thus represent a concept such as is involved in the word "nation," or the ideas represented by the adjective "beautiful." Dr. Wyllie's writings should stimulate other works in this important field to fill up some of these gaps, and will indicate along what lines advance may be made. The chief forms of aphasia, their mode of origin and their clinical characteristics, are fully considered; and more briefly the rarer and less well authenticated sub-varieties. With regard to the question why we are left-brained as regards speech, why the speech-centres are developed only in one hemisphere, the author adopts Dr. Moxon's explanation, which after all is not much more than a re-statement of the fact "that in the training of the speech-centres it is favourable to the operation of the educating attention that it should . . . be concentrated upon one hemisphere, rather than divided between two."

We cannot enter into the discussion of the many interesting points connected with aphasia, which the reader will find uniformly well treated in this book. In any individual case in which speech is regained, it is well to bear in mind one element of uncertainty in the diagnosis; namely, that it is impossible to be sure whether the recovery is due to the fact that the lesion of the speech-centre was only partial, or is to be attributed to the education of the opposite hemisphere. We may mention that the author regards the posterior end of the third frontal convolution—destruction of which is disastrous to speech in man, but whose stimulation produces no movement of the lips and tongue in animals—as a storehouse for guiding psycho-motor pictures or memories, made up of memories of muscular and tactile sensations, and the base of the ascending frontal and parietal convolutions as executive motor mechanisms, and that he, quite rightly as we think, sees no need to assume the existence of an "ideational" centre.

The concluding chapter describes the anatomy and physiology of the motor tracts for speaking and writing, and also gives an account of much practical utility of the pathological alterations that occur in them, and the symptoms produced by lesions at different levels. In the form of an appendix, three recent cases of aphasia are detailed, and the author's graduation thesis on the physiology of the larynx (1865), in which he points out the important action of the false vocal cords in closing the glottis, is reprinted.

In conclusion, we must note the excellent practical advice given throughout the book with regard to the diagnosis and treatment of speech disorders, especially in the section on stammering, the examination and treatment of deaf mutes and of otherwise healthy children brought for advice on account of inability to speak, and in the treatment of aphasia. That the book will fulfil the author's hopes in being "practically useful," and in proving "of practical value as an aid in the prosecution of this important study," we can have no manner of doubt.

The dress in which this work is presented to the reader, as regards binding, printing, &c., is in all respects good.

